# Editorial: Transcription factors as regulators of adaptive immunity

**DOI:** 10.3389/fgene.2026.1875399

**Published:** 2026-05-19

**Authors:** Liliana C. Patiño, Dunfang Zhang

**Affiliations:** 1 Department of Veterinary Medicine, Virginia-Maryland College of Veterinary Medicine, University of Maryland, College Park, MD, United States; 2 Department of Biotherapy, State Key Laboratory of Biotherapy, West China Hospital, Sichuan University, Chengdu, China

**Keywords:** adaptive immunity, antigen-presenting cells, autoimmunity, cancer, lymphocytes, transcription factors

Adaptive immunity involves pathogen-specific effector immune responses and specialized cells such as antigen-presenting cells (APCs) and lymphocytes. After their development, lymphocytes are activated by APCs to exert effector functions throughout the body (Chi et al., 2024). Successful immune responses result from the activation or inactivation of specific transcription factors in response to distinct signaling pathways. This Research Topic of papers, comprising two reviews and four original research articles, demonstrates how transcription factors are key regulators of adaptive immunity across a broad spectrum of diseases, including autoimmune disorders and cancer.

Autoimmune diseases are characterized by dysregulated cytokine expression that contributes to the maintenance of autoreactive lymphocytes. High levels of Interleukin-10 (IL-10) are associated with disease pathogenesis (Tian et al., 2014). c-Maf is a key transcriptional regulator that plays important role in suppressing the inflammation and promoting protective immunity through the modulation of IL-10. Liu et al. explore the mechanistic role of c-Maf and its associated signaling networks in adaptive immune cells such as B and T cells, as well as their synergy with innate immunity cells as macrophages. They describe how c-Maf disruption orchestrates the downregulation of other transcription factors including Blimp-1 and Stat3, leading to increased expansion of pro-inflammatory Th1 and Th17 cells in Inflammatory bowel disease (IBD) and systemic lupus erythematosus (SLE), or upregulating IL-21 in autoimmune diabetes. Additionally, c-Maf deficiency leads to dysfunction of intestinal RORγt^+^ regulatory T cells (Tregs) and type 1 regulatory T (Tr1) cells. The authors suggest the development of highly selective, disease-specific c-Maf modulators for autoimmune pathologies.

Glaucoma is a neurodegenerative disease of the optic nerve characterized by progressive degeneration of retinal ganglion cells and axons. Experimental models demonstrating optic nerve changes associated with immunoglobulin and T-cell infiltration, together with the detection of antibodies against heat-shock proteins in patients, provide evidence for an autoimmune component (Jiang et al., 2020). Wang et al. identified differentially expressed genes (DEGs) between open-angle glaucoma (POAG) and healthy individuals using public datasets. A total of 409 DEGs were identified using normalized expression data, including 267 upregulated and 142 downregulated genes. Analysis of immune infiltration revealed elevated levels of activated dendritic cells, natural killer cells, monocytes, and immature dendritic cells in glaucoma samples. To identify genes linked to the POAG phenotype, a gene co-expression network was constructed using the WGCNA algorithm, followed by functional enrichment analysis of immune-related genes associated with POAG. Three hub genes (*CD40LG*, *TEK*, and *MDK*) were identified, validated by qPCR, and proposed as potential diagnostic biomarkers for high-risk glaucoma patients.

Similarly, Li et al. evaluated the association of six SNPs in the *STAT4* gene with primary Sjögren’s syndrome (pSS) in Han Chinese women. This case–control study involved 269 female patients diagnosed with pSS and 594 healthy women. They demonstrate an association between the SNP rs10168266 (CC genotype) with increased pSS risk. Relative *STAT4* expression in PBMCs from healthy donors show a higher expression in rs10168266 CC carriers compared with CT and TT carriers, which correlated with elevated STAT4 and IL6 serum levels. This manuscript identifies a genetic determinant of pSS susceptibility in Han Chinese populations that may contribute to STAT4-mediated amplification of Th1/Th17 responses and IL-6 overproduction, offering a theoretical foundation for translational gene therapy.

Obesity and type 2 diabetes mellitus are strongly influenced by both genetic and environmental factors (Romao and Roth, 2008). The gut microbiota represents an additional component shaped by both. Stolarczyk et al. demonstrate that mice lacking the transcription factor T-bet are insulin sensitive. T-bet-deficient mice exhibit a higher proportion of colonic Tregs despite lower proportion of other lymphocytes populations compared with wild type (WT) mice. This alteration is accompanied by increased butyrate production, which is strongly linked to the development of peripheral Tregs and in the regulation of colonic Tregs homeostasis as consequence of the altered microbiota composition. They show that T-bet-deficient mice have changes in gut microbiota composition, with a greater proportion of Lachnospiraceae and fewer *Bacteroides* and *Prevotella* compared with WT mice. This study reveals an additional mechanism by which T-bet regulates colonic microbiota composition and gut immunity, influencing insulin sensitivity, suggesting that targeting T-bet axis may represent a novel therapeutic approach for insulin resistance.

In cancer, tumor-associated antigens activate the T cell response via major histocompatibility complex (MHC), representing an important line of defense against tumorigenesis (Dhatchinamoorthy et al., 2021). López-Santana et al. explore the relationship between key pluripotency-regulating transcription factors and immune responses in triple-negative breast cancer (TNBC). They highlight how signaling pathways governing stem cell differentiation—including Wnt/β-catenin, and TGF-β/SMAD pathways—are dysregulated in TNBC, contributing to immune evasion through mechanisms such as increased Treg activation, suppression of Th1, Th2, and NK cells, decreased antigen presentation, and production of the immunomodulatory cytokine IL-24. Additionally, the authors describe the role of pluripotency regulatory transcription factors including Oct4, Sox2, and c-Myc, and their involvement in MHC class I downregulation in TNBC.

ETV4, a member of the E26 transcription factor superfamily, is overexpressed in many tumor types including breast, prostate, and gastric cancers (Zhang et al., 2023). Huang et al. explore the prognostic value of ETV4 across various cancers, and its potential associations with immune-related molecules. They found that ETV4 overexpression is positively associated with cancer progression and may predict responses to immunotherapy. Using multiple public databases, they demonstrate that ETV4 expression is closely related to tumor cell proliferation, migration, and apoptosis, and significantly influences the expression of immune-related molecules, highlighting its potential impact on immunotherapy treatments, particularly in melanoma and kidney renal clear cell carcinoma (KIRC). Moreover, they validate a correlation between ETV4 and Fibrinogen-like protein 1 (FGL1), a major ligand for the inhibitory receptor LAG3, suggesting that the ETV4-FGL1 axis may be a key player in tumor immune evasion and may represent a potential target to enhance cancer immunotherapy.

In conclusion, transcription factors play essential roles in regulating gene expression in adaptive immune cells, including differentiation, activation, cytokine production, and environmental sensing (Patalano et al., 2025). Abnormal transcription factor expression can lead to dysregulation of immune signaling pathways and the development of immune-related diseases. The papers published in this Research Topic propose future therapeutic strategies for autoimmune diseases, —including glaucoma, pSS, obesity, and diabetes—as well as cancer, in which transcription factors are directly implicated.
